# Two novel compound heterozygous variants of *LTBP4* in a Chinese infant with cutis laxa type IC and a review of the related literature

**DOI:** 10.1186/s12920-020-00842-6

**Published:** 2020-12-10

**Authors:** Qiang Zhang, Zailong Qin, Shang Yi, Hao Wei, Xun Zhao Zhou, Jiasun Su

**Affiliations:** Laboratory of Genetic and Metabolism, Department of Paediatric Endocrine and Metabolism, Maternal and Child Health Hospital of Guangxi, Nanning, 530000 China

**Keywords:** ARCL IC, *LTBP4* gene, Mutation

## Abstract

**Background:**

Autosomal recessive cutis laxa type IC (ARCL IC, MIM: #613177) results from a mutation in the *LTBP4* gene (MIM: #604710) on chromosome 19q13.

**Case presentation:**

A 28-day-old Chinese infant with generalized cutis laxa accompanied by impaired pulmonary, gastrointestinal, genitourinary, retinal hemorrhage, abnormality of coagulation and hyperbilirubinemia was admitted to our hospital. To find out the possible causes of these symptoms, whole-exome sequencing was performed on the infant. Two novel pathogenic frame-shift variants [c.605_606delGT (p.Ser204fs * 8) and c.1719delC (p.Arg574fs * 199)] of the *LTBP4* gene associated with ARCL IC were found which was later verified by Sanger sequencing. The pathogenicity of mutations was subsequently assessed by several software programs and databases. In addition, an analytical review on the clinical phenotypes of the disease previously reported in literature was performed.

**Conclusions:**

This is the first report of a Chinese infant with ARCL IC in China due to novel pathogenic variations of *LTBP4*. Our study extends the cutis laxa type IC mutation spectrum as well as the phenotypes associated with the disease in different populations.

## Background

Cutis laxa, a disease with clinical heterogeneity, is characterized by inelastic and loose skin and it may present systemic manifestations of variable severity [1]. Autosomal recessive cutis laxa (ARCL) is comprise of three subtypes: ARCL I, (ARCL IA, ARCL IB and ARCL IC), and ARCL II (ARCL II A, ARCL IIB, ARCL IIC, ARCL ID and ARCL III [2]. Previous studies have revealed that LTBPS is related to ARCL IC in the gene encoding process [3]. To date, only twenty-four *LTBP4* variations have been recorded in a total of 20 sufferers. Herein, we uncovered two pathogenic variations in *LTBP4* by whole-exome sequencing (WES).

*LTBP4* is a key gene related to the formation of the microfibrillar structures [4]. Transforming growth factor beta (TGFβ) is secreted into the extracellular matrix as a tightly bound dimeric pro-peptide referred to as latency-associated peptide. This is the small latent complex, which then binds to *LTBP4*, to form a large latent complex. This process ensures that TGFβmolecules become a part of the microfiber system, from which the releasing process is managed by different mechanisms. Because of this, *LTBP4* has a crucial function in the regulation of TGFβ1 signaling [5, 6]. We were able to confirm that a Chinese newborn had signs of ARCL1 and summarized the characteristics of this individual as well as other patients with LTPB 4 related to cutis laxa.

## Case presentation

A 28-day-old Chinese newborn was admitted to our medical center for jaundice. According to her initial examination report, skin laxity and wrinkling were observed and the patient was suspected of having cutis laxa. Therefore, she was subjected to further physical examination and testing, including radiographic, genetic and routine biochemical tests.

## Genetic analysis

Genomic DNA extraction (Lab-Aid DNA kit, Zeesan Biotech Co., Ltd. China), which target capturing (Human All Exon V5 Kit, Agilent Technologies, CA) and library sequencing (Hiseq 2500 platform, Illumina, USA) were conducted by using standard manufacturers’ protocols of kits. Data analysis and annotation were performed based on the Genome Analysis Toolkit. A causal variant was identified by bioinformatics screening (TGex, LifeMap Sciences, USA). Finally, the mutations were tested and verified by Sanger sequencing and their pathogenicity were assessed using the ACMG/AMP 2015 guidelines [7].

## Clinical characteristics of the patients with ARCL IC

**Fig. 1:**
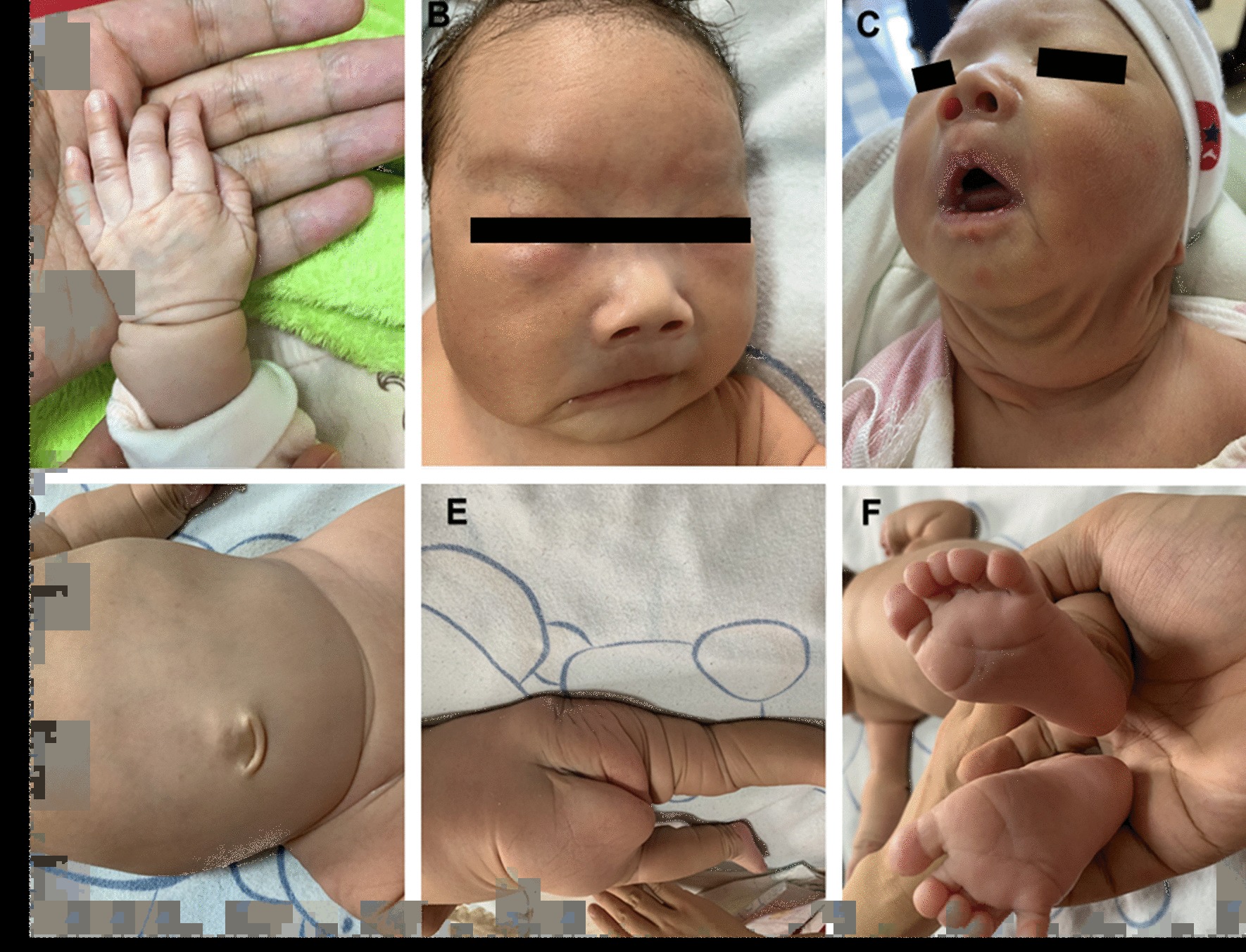
The clinical features of the proband with Cutis laxa. **a** Shows the infant’s hand with a thinning of the skin and visible veins; **b** shows dysmorphic facial features; **c**, **e**, **f** show thinning of the skin with poor elasticity, dryness, as well as small wrinkles on the bottom of feet; **d** shows an umbilical hernia

**Fig. 2:**
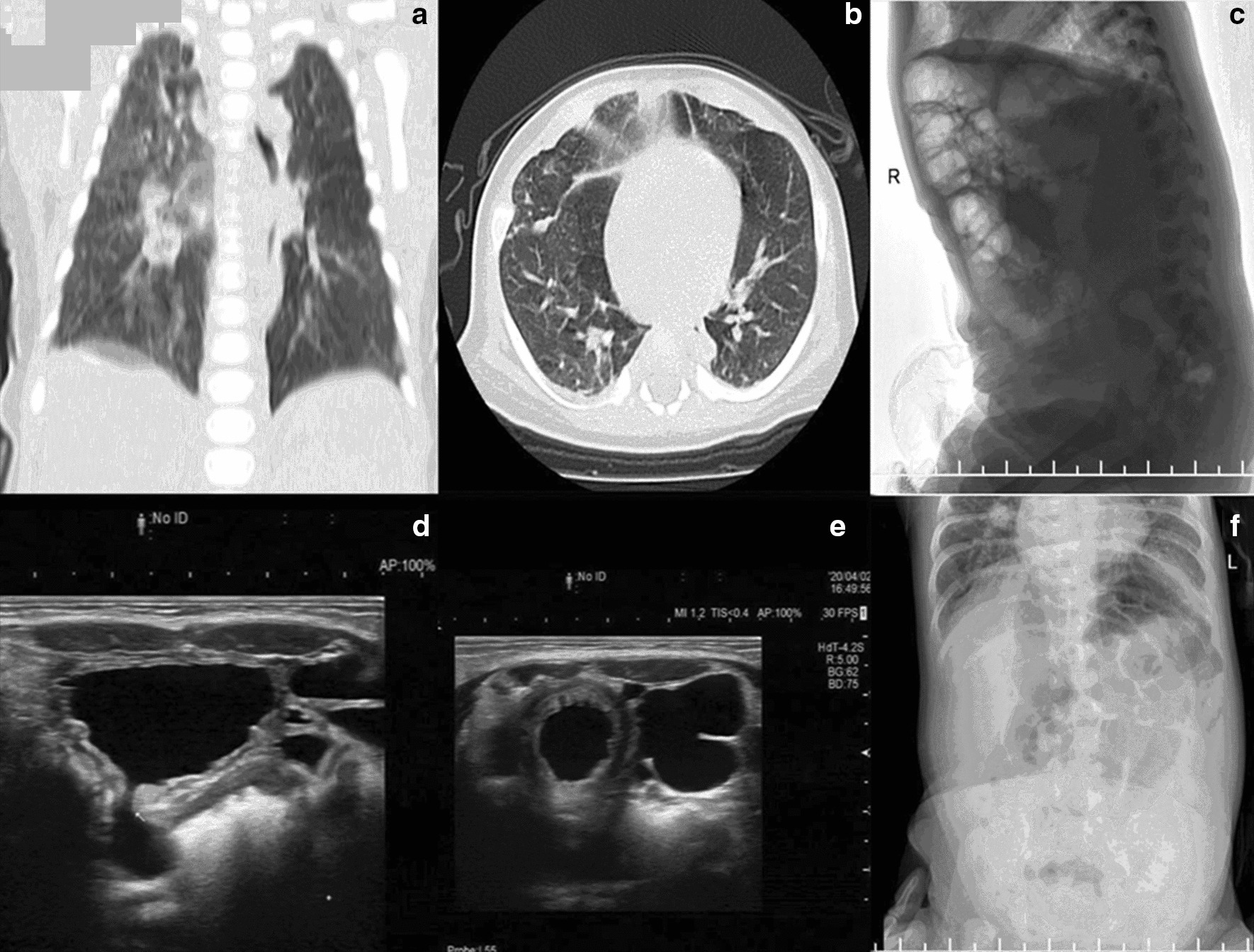
Clinical examinations of the patient. **a**, **b** Shows bilateral pneumonia and emphysema; **c**, **f** shows dilatation of the intestine; **d**, **e** shows multiple diverticulum of bladder

The propositus was from Nanning, Guangxi, China and she admitted to our hospital due to neonatal jaundice. She was the third-born child of a healthy and non-consanguineous couple at 36^+2^ weeks gestation. The infant was breeched at birth and weighed 3.02 kg (P50–P90) with a height of 50 cm high (> P90) and with her head and chest circumference measurements were 34 cm (P50–P90) and 32 cm (P50), respectively. The patient had no history of asphyxiation after birth. Physical examination showed that she had dysmorphic facial features including a fat midface, smooth philtrum, hypertelorism and downturned corners of the mouth. Her skin appeared dry with poor elasticity and thinning as well as visible veins along with small wrinkles on the limbs (Fig. 1). However, she appeared to have normal muscle force and muscle strength. Ultrasound examination revealed that the liver, cranial, articulatio coxae and genital system were normal. Nonetheless, imaging examination of the lungs (bilateral pneumonia and emphysema), kidneys (multiply bladder diverticulum) and intestines (dilatation of intestine) suggested some abnormalities (Fig. 2). The test using heart color ultrasound only indicated a patent foramen ovale (2 mm), and the Doppler ultrasonography images of the aorta and liver were normal.

Some biochemical tests were performed on the patient and the results showed an increase in the levels of serum total bilirubin (225.47 umol/L, 3.00–22.00 umol/L), direct bilirubin (9.59 umol/L, 0.00–6.80 umol/L), creatine kinase (379.00 u/L, 25.00–200.00 u/L), creatine kinase isoenzyme (32.00 u/L, 0.00–25.00 u/L), lactic acid (4.8 mmol/L, 0.50–1.60 mmol/L) and aspartate aminotransferase (AST, 50 u/L, 4.00–40.00 u/L) as well as the fibrinogen index (PT: 13.2 s, 8.80–11.80 s; INR: 1.22, 0.83–1.12; APTT: 49.2 s, 28.00–41.00 s; TT: 17.9 s, 10.30–16.60 s; DD: 1530 ng/mL FEU, 0.00–550.00 ng/mL FEU). However, results of blood gas analysis suggested that acidosis had occurred (pH: 7.31, 7.35–7.45; SBE: − 7.0 mmol/L, − 3.00 to 3.00 mmol/L). Blood routine examination showed WBC (6.4 * 10^9/L, 4.00–10.00 * 10^9/L), RBC (4.5 * 10^12/L, 3.50–5.00 * 10^12/L) and HB (156 g/L, 110.00–150.00 g/L) were normal, but with increases in the percentages of lymphocytes (Lym%, 42.6%, 20.00–40.00%) and reticulocytes (6.01%, 0.50–2.50%).

**Fig. 3:**
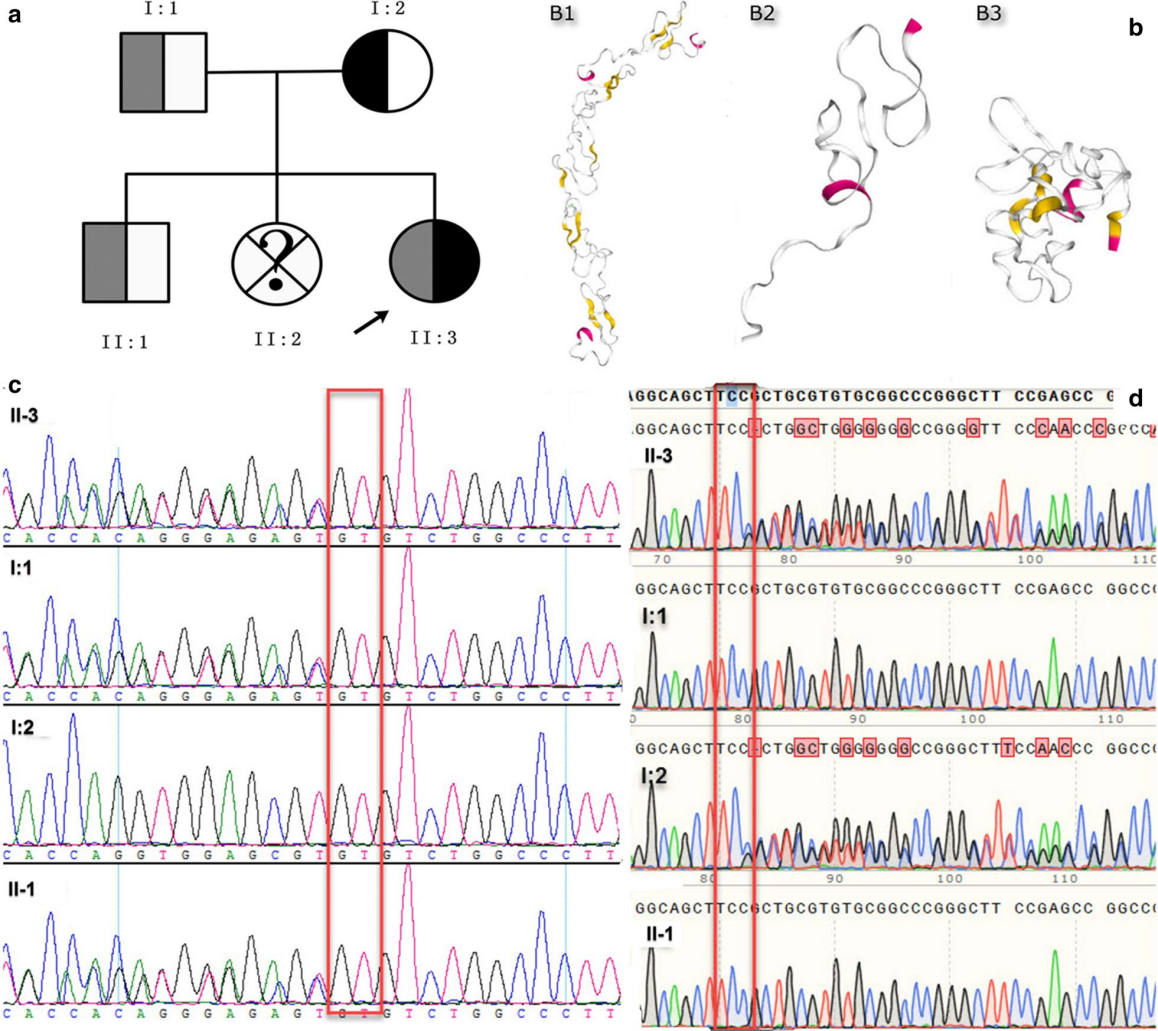
**a** Pedigree with cutis laxa type IC. Filled symbols represent affected individuals, open symbols unaffected individuals, semi filled represent carriers, squares depict males and circles depict females, crisscrossing lines indicate deceased individuals, a question mark means the cause of death remains unknown and the proband is indicated by an arrow. **b** The three-dimensional structure modeling predicts a decrease in protein length with B1 as the wild-type and B2 and B3, the mutant-types (B2: p.Ser204fs * 8; B3:p.Arg574fs * 199). **c**, **d** Sanger sequencing chromatograms showing variations in the affected individuals in comparison to those of unaffected individuals (**c** is the sequencing result of c.605_606delGT and **d** is the sequencing result of c.1719delC), II:1 is elder brother, II:2 is the second elder sister, II:3 is proband and I:1 is father, I:2 is mother

The results of hearing screening tests revealed no abnormalities. However, the eyes screening results indicated that the patient had a subhyaloid hemorrhage. By enquiring into her family’s medical history, we found that the mother had delivered a baby girl with similar clinical symptoms in 2012, who kept vomiting and bloating for 2 months after birth. At that time, doctors diagnosed that she had intestinal necrosis and advised her to undergo surgical treatment. Soon after that episode, the baby died and her etiology remains unknown (Fig. 3a).

## Bioinformatic analysis and verification of observations

WES was performed and 18.1G clean data were generated covering 95.5% of exome target regions at minimum of 20X. As a consequence, the exome sequencing had interrrogated a total of 135,155 functional variants. Assisted by TGex software, we successfully selected 9 variants included in the OMIM gene whose functions matched the patient’s phenotypes as candidate variants. Basically, we considered compound heterozygous in the *LTBP4* gene shared the highest probability. Both c.605_606delGT (p.Ser204fs * 8) and c.1719 del C (p.Arg574fs * 199) were not presented in 1 K Genomes (https://www.internationalgenome.org/), HGMD (http://www.hgmd.cf.ac.uk/ac/), ClinVar (https://www.ncbi.nlm.nih.gov/clinvar/) and LOVD (http://www.LOVD.nl/LTBP4).

Sanger sequencing was then applied to verify the variants using the following forward and reverse primers: 5′ CCCGTAAGAACCCGTGTAGA 3′ and 5′ CAGCTCCCGAAAGCAGTAAC 3′ for c.605_606delGT/p.Ser204fs * 8 and 5′ CTTTGCCTGGTCACCTTGTC 3′ and 5′ AGTGAGAGCAGGAGCGAGAC 3′ for c.1719delC/p.Arg574fs * 199. In accordance with the results of Sanger sequencing, the former mutation was found to be inherited from the father, while the latter was inherited from the mother (Fig. 3c, d). Following this, the protein-structure of the WT (wild type) and mutant *LTBP4* were predicted by SWISS-MODEL (https://swissmodel.expasy.org/interactive). This results indicated that the mutations changed the size of protein from 1074 to 542 (c.1719delC/p.Arg574fs * 199) and 146 amino acids (c.605_606delGT/p.Ser204fs * 8). These mutations caused the open reading frame (ORF) of *LTBP4* to be terminated prematurely. This resulted in the overall shape of *LTBP4* protein to be subsequently altered as shown in Fig. 3b (B1, B2 and B3).

## Discussion and conclusions

Cutis laxa type IC is usually the result of homozygous or compound heterozygous mutations in the *LTBP4* gene located on chromosome 19q13.1-19q13.2. The disorder is considered to be extremely rare as its existence has only been confirmed in 19 families. However its etiology and prevalence rate remains unclear. The size of *LTBP4* mRNA is about 5.1 kilobase pairs and it is primarily expressed in the aorta, small intestine, uterus and heart [8].

Immunoblotting studies confirmed that cultured human lung fibroblasts can secrete *LTBP4*, a protein which belongs to a family of four extracellular matrix proteins that are structurally associated with fibrillins [9]. It consists of epidermal growth factor-like domains interspersed with four 8-cys domains. The third 8-cys domain can bind to the latent complex which consists of a TGFβ1 homodimer together with its pro-peptide so as to sequester TGFβ1 and orchestrate its activation [4, 10]. Additionally, with the aid of regulating the incorporation of elastin-fibulin-5 complexes into the microfibrillar bundles to form elastic fibers, *LTBP4* may enhance the process of elastogenesis [4].

Recent evidence suggests that *LTBP4* helps to stabilize both the TGF β receptors, TGFBR1 and TGFBR2 [11]. The loss of *LTBP4* could, therefore, lead to decreased TGFβsignaling in skin fibroblasts which is caused by rapid degradation of TGFBR1/TGFBR2 receptor complexes [13]. Therefore, *LTBP4* is a critical component for the normal growth and development of human internal organs. Mutations in *LTBP4* will lead to a severe condition referred to as LTBP deficiency syndrome. As can be seen clearly from the comprehensive review of the literature (Table 1), cutis laxa caused by *LTBP4* shows loosening of the skin on the trunk and limbs and droopy as well as puffy facial skin. These symptoms appear from the birth in all patients (21/21, 100%), while the clinical manifestation of impaired pulmonary functions usually occurs in the first month of life. Thus it considered a severe disease and it can usually be fatal due to the aggravating factors which may include emphysema (16/17, 94.12%), respiratory distress (17/19, 89.47%) and pneumonia (7/11, 63.64%).

**Table 1:** Summary of clinical features of all patients with *LTBP4* pathogenic variants

Citations	This report	P1 [2]	P2 [3]	P3 [3]	P4 [3]	P5 [3]	P6 [10]	P7 [10]	P8 [10]	P9 [10]	P10 [10]	P11 [10]	P12 [10]	P13 [10]	P14 [10]	P15 [11]	P16 [11]	P17 [11]	P18 [11]	P19 [12]	P20 [12]	Ration
Gender/age	F/ 28D	F/ 18MO	M/ 9MO	M/ 4MO	F/ 7Y	F/ 26MO	F/ 23Y	M/ 4W	F/ 3MO	M/ 2Y	M/ 10Y	M/ 6MO	F/ 6MO	F/ 13Y	M/ 6W	M/ 15MO	F/ 14Y	F/ 20Y	M/ 6W	F/ 8Y	M/ 4Y	M/F:1.1:1 4.74 ± 6.95
Country/Race	CN	VEN	ESP	MEX	PLE	ESP	NA	NA	NA	NA	NA	NA	NA	NA	NA	White	White	White	TUR	IN	IN	–
Cutis laxa	+	+	+	+	+	+	+	+	+	+	+	+	+	+	+	+	+	+	+	+	+	100.00%
Joint laxity	+	+	+	+	+	+	+	−	−	−	−	−	−	−	+	NA	+	+	+	−	−	55.00%
Craniofacial																						
Consanguinity	−	+	+	−	+	−	−	+	−	+	+	+	+	+	+	−	−	−	+	−	−	52.38%
Long philtrum	+	+	+	+	+	NA	−	NA	NA	+	NA	NA	+	+	−	−	+	+	NA	+	+	80.00%
Fat midface	+	+	+	+	+	NA	−	NA	NA	−	NA	NA	−	−	−	−	NA	NA	NA	+	+	53.85%
Narrow forehead	+	+	+	+	NA	+	−	NA	NA	+	NA	NA	+	+	+	−	+	NA	NA	+	+	85.71%
Periorbital swelling	−	+	+	+	NA	NA	−	NA	NA	+	NA	NA	+	−	−	+	NA	NA	NA	NA	NA	60.00%
Hypertelorism	+	+	+	+	NA	NA	−	NA	NA	+	NA	NA	+	+	−	+	+	+	+	NA	NA	84.62%
Depressed nasal bridge anteverted nares	+	+	+	+	NA	NA	−	NA	NA	+	NA	NA	+	−	+	+	+	NA	NA	NA	NA	81.82%
Micrognathia	+	+	+	+	+	+	−	NA	NA	−	NA	NA	−	−	−	−	NA	NA	NA	+	+	57.14%
Pulmonary																						
Respiratory distress	+	+	+	+	+	+	+	+	+	+	+	+	+	+	+	+	+	NA	NA	−	−	89.47%
Pneumonia	+	+	+	−	−	+	NA	NA	NA	NA	NA	NA	NA	NA	NA	+	+	NA	+	−	−	63.64%
Laryngomalacia tracheomalacia bronchomalacia	+	−	−	+	NA	+	−	+	−	−	−	−	−	−	+	−	NA	NA	NA	−	−	29.41%
Diaphragmatic hernia or eventration	−	+	−	+	+	+	+	−	−	+	+	−	+	−	+	+	NA	NA	NA	−	−	55.56%
Emphysema	+	−	+	+	NA	+	+	+	+	+	+	+	+	+	+	+	NA	NA	NA	+	+	94.12%
Gastrointestinal																						
Diverticula	−	−	−	+	+	NA	−	−	−	−	−	−	+	−	−	+	NA	NA	+	−	−	27.78%
Intestinal dilation	+	+	+	+	NA	−	−	−	−	−	−	+	−	−	−	+	NA	NA	+	−	−	38.89%
Rectal prolapse	−	−	−	−	+	+	−	−	−	−	+	−	−	−	−	NA	NA	NA	NA	−	−	17.65%
Genitourinary																						
Bladder diverticula	+	−	+	+	+	NA	+	−	+	−	+	−	−	+	+	+	NA	−	+	+	+	68.42%
Hydronephrosis	−	+	+	+	−	NA	−	+	−	−	−	−	−	−	+	NA	NA	NA	NA	−	+	37.50%
Inguinal hernia	−	−	+	+	−	−	−	−	−	−	+	−	−	−	−	+	NA	NA	NA	−	−	22.22%
Cardiovascular																						
Peripheral pulmonary artery stenosis	−	−	+	−	NA	+	+	+	−	+	−	−	+	+	−	+	+	+	+	−	−	55.00%
Atrial septal defect or aneurysms	−	+	−	−	NA	−	−	−	−	−	+	−	+	−	−	−	−	+	+	−	+	30.00%
Cardiac valve insufficiency	−	−	−	−	NA	−	−	−	−	NA	+	−	+	−	+	+	+	−	−	−	−	26.32%
Pulmonary or aortic valve stenosis	−	−	−	−	NA	+	−	−	−	−	−	−	−	−	−	+	−	−	−	−	−	10.00%
Pulmonary hypertension	−	−	+	−	NA	+	−	−	−	−	−	+	+	+	+	+	−	+	−	−	−	40.00%
Patent foramen ovale	+	−	+	+	−	NA	−	−	−	−	−	−	−	−	+	+	−	−	−	−	−	25.00%
Hypotonia	−	+	+	+	NA	+	−	+	−	+	−	−	+	−	+	+	NA	NA	+	+	−	61.11%
Death or not	−	−	+	+	−	+	−	+	+	+	+	+	+	+	+	NA	−	−	+	−	−	60.00%

*CN* China, *VEN* Venezuelan, *ESP* hispanic, *MEX* Mexican, *PLE* Palestinian, *TUR* Turkish, *IN* India, *NA* not available, *F* female, *M* man, *D* day, *Y* year, *MO* month

In humans and animal models with *LTBP4* deficiency it was shown that emphysema was caused by impaired terminal air sac septation [14, 15]. The gastrointestinal can also be affected and problems may include the following: intestinal dilation (7/18, 38.89%), diverticula (5/18, 27.78%) and rectal prolapse (3/17, 17.65%). Tracing back to the proband’s family history, her elder sister died from suspected digestive problems. Genitourinary symptoms are quite common and these can be more noticeable over time, the root of which may be in hydronephrosis (6/16, 37.50%), bladder diverticula (13/19, 68.42%) and inguinal hernia (4/18, 22.22%). Besides, some patients are also at risk for cardiovascular problems, including peripheral pulmonary artery stenosis (11/20, 55.00%) and patent foramen ovale (5/20, 25.00%). 61.11% of the patients showed hypotonia (11/18) which was believed to be the reason for motor development delay. As for the growth delay symptoms, these only occurred in a small number of patients and some scholars speculate that these are secondary symptoms and not related to *LTBP4*. There is a lack of written records with respect to the patients’ medical histories and this makes it extremely difficult to extend our studies of this disease as most of patients die before language development.

According to the Table 1, we can conclude that the core clinical symptoms of LTBP deficiency syndrome are cutis laxa, respiratory distress, emphysema and facial abnormalities, while the others belong to the heterogeneous manifestations. Some patients appear to have a few clinical characteristics which has never been reported in cutis laxa type IC, such as retinal hemorrhage, abnormality of coagulation and hyperbilirubinemia. We speculate that the hyperbilirubinemia may be related to premature delivery (36^+2^ weeks), acidosis (lactic acid: 4.8 mmol/L, pH: 7.31) and pulmonary infection, which can all affect bilirubin metabolism, and therefore, induce an increase in unconjugated bilirubin. The patients tend to have a poor prognosis, with a high mortality rate (60.00%, 12/20).

**Fig. 4:**
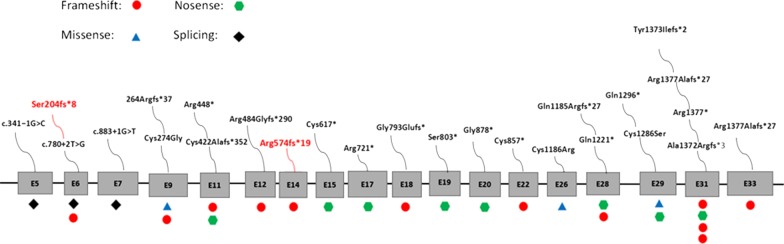
*LTBP4* pathogenic variants. This is the first report of Ser 204fs * 8 and Arg574fs * 199 frame-shift variants in this gene

The majority of the current descriptions of the pathogenic variants (Fig. 4) is frame-shift (10/24), nonsense (8/24), missense (3/24) and splice variants (3/24). This study reports on a 28-day-old girl with compound heterozygous variants of *LTBP4*, c.605_606delGT (p.Ser204fs * 8) and c.1719 del C (p.Arg574fs * 199), both of which are frame-shift mutations. The first is the 8th amino acid after the mutation and it becomes a termination codon, while the second is the 199th amino acid. They can both cause protein translation to terminate prematurely. It is generally believed that in a frame-shift mutation, if the error code formed includes a stop codon, the peptide chain will be shortened, resulting in a premature termination codon and activation of the nonsense-mediated mRNA decay (NMD). After the frame-shift mutation occurs, the activity of the protein encoded by the gene changes greatly [16–18], so it is very likely to be a lethal mutation. In a subsequent follow-up, we found that the patient’s respiratory distress was getting worse. Unfortunately, due to the fact that the parents had a previous child with similar symptoms (II-2), they anticipated the worse for the disease and treatment process, which led to their decision of not pursuing treatment for their child.

The identification of two novel mutations in the *LTBP4* gene make this the first report of this disease in China. Both medical symptoms and the mutations reported in the study can extend mutation spectrum and enrich the phenotype spectrum of cutis laxa IC in different populations. In addition, the study proves the potential value of mutation-based genetic screening and diagnosis within the future.

## Data Availability

The data that support the findings of this study are available from the corresponding.

## Abbreviations

ARCL IC: Autosomal recessive cutis laxa type IC
WES: Whole exome sequencing
TGFβ: Transforming growth factor-beta
LTBPs: Latent TGFβ binding proteins
NMD: Nonsense-mediated decay
ORF: Open reading frame
SBE: Standard base excess
